# Genetic and Environmental Dispositions for Cardiovascular Variability: A Pilot Study

**DOI:** 10.3390/jcm7090232

**Published:** 2018-08-23

**Authors:** Radmila Karan, Suzana Cvjeticanin, Natasa Kovacevic-Kostic, Dejan Nikolic, Milos Velinovic, Vladimir Milicevic, Biljana Obrenovic-Kircanski

**Affiliations:** 1 Faculty of Medicine, University of Belgrade, 11000 Belgrade, Serbia; cujasimsi@gmail.com (S.C.); cipelcici@yahoo.com (N.K.-K.); denikol27@gmail.com (D.N.); velinovicsurg@gmail.com (M.V.); biljanaok@yahoo.com (B.O.-K.); 2 Department of Anesthesiology and Intensive Care, Clinic for Cardiac Surgery, Clinical Center of Serbia, Koste Todorovica 8, 11000 Belgrade, Serbia; 3 Institute for Human Genetics, Faculty of Medicine, University of Belgrade, 11000 Belgrade, Serbia; 4 Department of Physical Medicine and Rehabilitation, University Children’s Hospital, 11000 Belgrade, Serbia; 5 Clinic for Cardiac Surgery, Clinical Center of Serbia, 11000 Belgrade, Serbia; vladodrillwork@gmail.com; 6 Clinic for Cardiology, Clinical Center of Serbia, 11000 Belgrade, Serbia

**Keywords:** coronary artery disease, homozygously-recessive characteristics, risk factors, variability, blood groups

## Abstract

Background: The aim of our study was to evaluate the degree of genetic homozygosity in the group of patients with coronary artery disease (CAD), as well as to evaluate morphogenetic variability in CAD patients regarding the presence of investigated risk factors (RF) compared to a control sample of individuals. Additionally, we aimed to evaluate the distribution of ABO blood type frequencies between tested samples of individuals. Methods: This study analyzed individual phenotype and morphogenetic variability of 17 homozygously-recessive characteristics (HRC), by using HRC test in a sample of 148 individuals in CAD patients group and 156 individuals in the control group. The following RF were analyzed: hypertension, diabetes mellitus, hyperlipidemia, and smoking. Results: The mean value of HRC in CAD patients is significantly higher, while variability decreases compared to the control sample (CAD patients: 4.24 ± 1.59, control sample: 3.75 ± 1.69; V_CAD-patients_ = 37.50%, V_C_ = 45.07%). There is a significant difference in individual variations of 17 HRC between control sample and CAD patients (*χ*^2^ = 169.144; *p* < 0.01), which points out to different variability for tested genes. Mean values of HRC significantly differed in CAD patients in regard to the number of RF present. A blood type (OR = 1.75) is significant predictor for CAD, while O blood type (OR = 0.43) was significantly associated with controls. Conclusion: There is a higher degree of recessive homozygosity in CAD patients versus individuals in the control sample, and the presence of significant variations in the degree of recessive homozygosity as the number of tested RF increases.

## 1. Introduction

Coronary artery disease (CAD) is not only the leading cause of mortality in highly-developed countries, but also a major cause of working disability and invalidity as well. Many recent studies agree that etiology of CAD is of multifactorial origin. In the last 2–3 decades, it was established that heredity of CAD with acute myocardial infarction (AMI) could be autosomal-dominant (OMIM number 608320 15q26.3). The genes, so far known to be involved in determination of susceptibility to CAD are 2q21.1-q22 (OMIM number 608316), 16pter-p13 (OMIM number 607339), 14q32 (OMIM number 608318), 8p22 (OMIM number 612030), and some studies have also suggested X-linked inheritance Xq23-q26 (300464) [1,2].

So far, there are numerous known risk factors for CAD. Among them are smoking, inadequate nutrition, obesity, diabetes, sedentary lifestyle, and hypercholesterolemia, and they are determined by gene loci [1,3,4,5].

Published scientific literature shows a connection between genetic predisposition for developing risk factors for CAD, as well as CAD itself. This inheritance can be either autosomal-dominant [6], autosomal-recessive, or linked to X chromosome [7].

The use of homozigously-recessive characteristics (HRC) test, used to determine genetic homozygosity in humans over 30 years, has shown that the group of individuals with different conditions have higher genetic homozygosity and lower variability for tested genes compared to the healthy control groups [8,9,10,11].

Recent findings stressed that the distribution of ABO blood types differ in samples of patients affected by different diseases and conditions [9,12]. 

Being that previous studies have shown that CAD is a genetically determined condition, as well as the fact that the number of HRC represent recessive homozygosity and is basically an estimation of genetic loads present in human population [11,13,14,15,16], we hypothesized that changed genetic homozygocity and variability in the affected individuals with CAD could, to the certain degree, correlate with the development of CAD. Additionally, previous studies have demonstrated that the frequencies of ABO blood types differed in different samples of patients [17,18,19], thus, we hypothesized that patients with CAD could have different frequencies distribution of ABO blood types.

Therefore, our aim was two-fold. First, the aim of our study was to evaluate degree of genetic homozygosity in patients with CAD, as well as to evaluate morphogenetic variability in CAD patients regarding the presence of studied risk factors compared to the control sample of individuals. The second aim was to evaluated distribution of ABO blood type frequencies between tested samples of individuals.

## 2. Materials and Methods

### 2.1. Study Group

In this cross-sectional study we evaluated 148 patients that were diagnosed with coronary artery disease (CAD patients) and referred to Clinic for Cardiac Surgery for on-pump surgical treatment between 2015 and 2017. Additionally, as the control we evaluated 156 individuals without CAD (control sample) during the same period of time. Both groups of individuals belong to the same ethnic group (Serbian population) and from the similar social-economic background and were between 56 and 65 years of age. The individuals from the control sample were chosen randomly by computer (method of simple random sampling) from the local Health care facilities in three cities, representing three regions of Serbia (Nis (central and southern Serbia)—up to 52 individuals, Belgrade (capital territory)—up to 52 individuals, and Novi Sad (Vojvodina)—up to 52 individuals). Prior enrollment into the study patients were explained the study protocol and signed informed consent was obtained. Study followed the principals of good clinical practice and was approved by relevant Institutional Review Boards of Clinical Center of Serbia, and Faculty of Medicine, University of Belgrade. 

For the confirmation of CAD diagnosis referred individuals were screened and evaluated by Board certified cardiologist, cardiac surgeons and radiologist. Both CAD patients and the control sample were screened for risk factors: hypertension (HTN), diabetes mellitus (DM), hyperlipidemia (HLP), and smoking.

### 2.2. Study Methods

Homozygous recessive characteristics (HRC) test (HRC-test) was used to evaluate the degree of recessive homozygosity and variability in tested groups of individuals. The performed test was comprised of 17 HRCs, where only characteristics with extreme appearance were marked as a present trait. The HRC-test has been developed to establish the proportion of homozygously-recessive clearly-expressed characteristics in every individual as markers of chromosomal homozygosities, implicating the degree of genetic homozygosity in humans [8,16,20,21]. It is reliable method for estimating the individual homozygosity and is performed by direct observation of defined phenotype traits [9,10,16,20,21,22]. To secure the same objectivity and equal criteria for determination of HRCs, one person performed the testing on studied population.

Hypertension was defined as a systolic blood pressure (SBP) ≥ 140 mm Hg, or a diastolic blood pressure (DBP) ≥ 90 mm Hg [23]. Diabetes mellitus was defined as fasting plasma glucose level (FPG) ≥ 7.0 mmol/L, or random plasma glucose level ≥ 11.1 mmol/L [24]. 

Hyperlipidemia was defined as total cholesterol levels > 5.17 mmol/L; LDL-C > 2.5 mmol/L in individuals without CAD or any risk factors; LDL-C > 1.8 mmol/L in individuals with risk factors or established CAD [25,26]. Patients referred to us for surgical procedures already had established diagnosis of hyperlipidemia by either an endocrinologist or cardiologist, who diagnosed the condition and set the course of treatment according to the American Heart Association guidelines for the management of hyperlipidemias [26]. Smoking was established by direct yes or no question that was formulated in a way that could be easily understood in the given questioner (Do you currently smoke?).

Considering the number of RFs, study groups were divided into: group with no RF (0 RF); group with one RF (1 RF); group with two RFs (2 RF); group with three RFs (3 RF), and group with 4 RFs (4 RF).

### 2.3. Tested Determinants 

We have evaluated 17 HRCs in every studied individual: attached ear lobe (OMIM number 128900), blue eyes (gene location 15q12, 15q13, OMIM number 227220; 5p13 OMIM number 227240; 14q32.1, OMIM number 210750; 9q23 OMIM number 612271), straight hair (1q21.3, OMIM number 139450), soft hair and blond hair (gene location 15q12, 15q13, OMIM number 227220; 14q32.1, OMIM number 210750; 12q21.3 OMIM number 611664; 11q13.3, OMIM number 612267), double hair whorl, opposite hair whorl orientation (OMIM number 139400), as well as an inability to roll, fold, and curve the tongue (OMIM number 189300), ear without Darwinian notch, and a guttural “r” [21,27], proximal thumb hyperextensibility, index finger longer than the ring finger (OMIM number 136100), left-handedness (gene location 2p12-q22, OMIM number 139900), right thumb over left thumb (hand clasping) (OMIM number 139800), and top joint of the thumb >45° [27]. Considering the tested ABO blood types and the pattern of inheritance, both A and B alleles are considered as codominant, and O allele as recessive [27].

### 2.4. Statistical Analysis

HRC frequencies in the control sample and CAD patients were presented as whole numbers and percentages, while average proportions of HRCs were represented as mean values (MV) and standard deviation (SD). The chi-squared test (*χ*^2^) was performed to compare frequencies of HRC between control sample and CAD patients, and tested parameters (gender, HTN, DM, HLP, and smoking) between control sample and CAD patients. The variation coefficient (V) was used to compare variability between evaluated groups of individuals. Comparison of HRC mean values regarding the number of RF between CAD patients and control samples was done by the Mann-Whitney U test. Unifactorial ANOVA test, and the two-way ANOVA were used to assess the statistical significance among HRC frequencies in the controls and CAD patients in regard to the presence and number of risk factors. To evaluate significant predictors of number of HRCs between tested groups of individuals, and between controls and CAD group for ABO blood types we used odds ratio (OR). Statistical significance was set on *p* < 0.05.

## 3. Results

In Table 1, demographic and RF frequencies are presented. There is no significant difference between genders for tested groups of individuals (for males *χ*^2^ = 3.605; *p* > 0.05, for females *χ*^2^ = 3.798; *p* > 0.05), same applies for HLP (*χ*^2^ = 0.877; *p* > 0.05), and smoking frequencies (*χ*^2^ = 1.899; *p* > 0.05) (Table 1). Significant difference was noticed in frequencies between tested groups for HTN (*χ*^2^ = 27.664; *p* < 0.01), and DM (*χ*^2^ = 59.415; *p* < 0.01) (Table 1).

In Table 2, distribution of HRC frequencies both for the control sample and CAD patients were presented. There were nine HRCs that significantly differed, of which six (ear without Darwinian notch, blue eyes, speaking deficiency guttural “r”, inability to transversally tongue roll, inability to longitudinally tongue roll, and proximal thumb extensibility) were significantly more frequent in CAD patients, while three (double hair whorl, top joint of the thumb >45°, and index finger longer than the ring finger) were remarkably higher in control sample. There is significant difference in individual variations of 17 HRCs between control sample and CAD patients (∑*χ*^2^ = 169.144; degree of freedom (df) = 16, *p* < 0.01) (Table 2). The most significant predictor for CAD is HRC-ear without Darwinian notch (OR-4.45) (Table 2).

**Figure 1:**
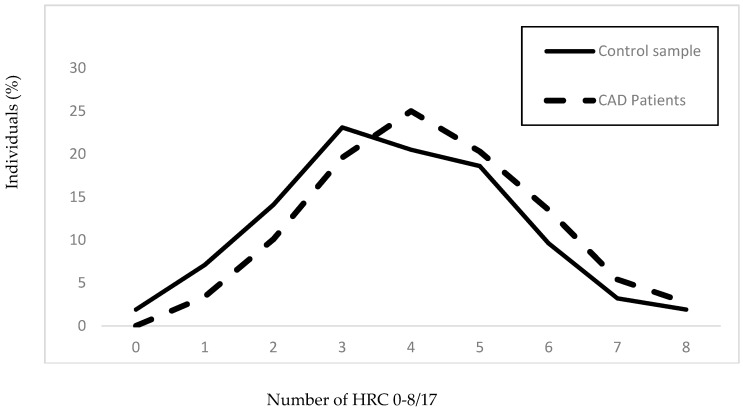
Frequencies of homozygous recessive characteristics in the group of patients with CAD and the control sample. Control sample: *N* = 156, x_hrc/17_ = 3.75 ± 1.69; CAD patients: *N* = 148, x_hrc/17_ = 4.24 ± 1.59 (*z* = −2.498, *p* < 0.012); V_Control sample_ = 45.07%, V_CAD patients_ = 37.50%.

In Figure 1, it is shown that the mean value of HRC in the evaluated sample of CAD patients is significantly higher, while variability decrease comparing to the control sample (control sample _(MV±SD)_: 3.75 ± 1.69, CAD patients_(MV±SD)_: 4.24 ± 1.59; V_Control sample_ = 45.07%, V_CAD patients_ = 37.50%).

**Figure 2:**
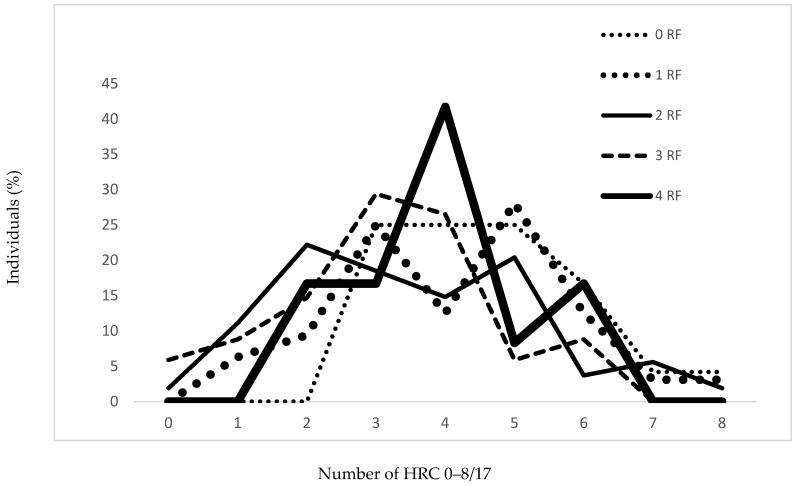
Frequencies of homozygous recessive characteristics in the control sample regarding number of risk factors. RF: risk factor; 0 RF: *N* = 24 (15.4%); 1 RF: *N* = 32 (20.5%); 2 RFs: *N* = 54 (34.6%); 3 RFs: *N* = 34 (21.8%); 4 RFs: *N* = 12 (7.7%).

In Table 3 and Figure 2, it is shown that there is significant difference among frequencies of HRCs in regard to the presence and number of risk factors for the controls (*p* = 0.007) (Table 3). However, variability in the control sample tends to increase with regard to the presence of risk factors with the peak at 2 RF, after which it declines (V_0 RF_ = 29.81%, V_1 RF_ = 39.47%, V_2 RF_ = 52.30%, V_3 RF_ = 48.89%, V_4 RF_ = 33.42%) (Table 3 and Figure 2).

**Figure 3:**
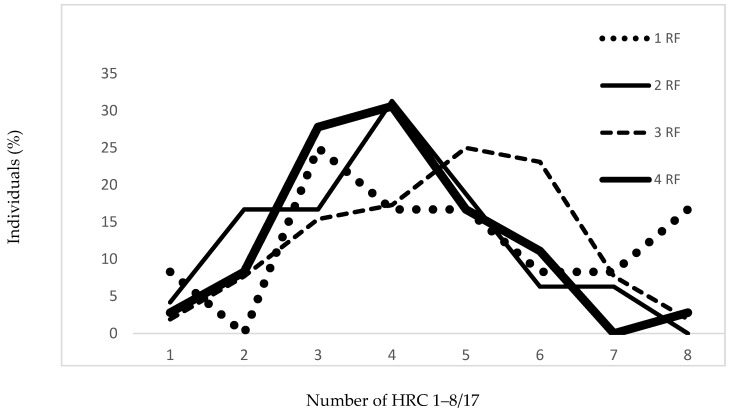
Frequencies of homozygous recessive characteristics in CAD patients regarding number of risk factors. RF: Risk factors; 1 RF: *N* = 12 (8.1%); 2 RFs: *N* = 48 (32.4%); 3 RFs: *N* = 52 (35.2%); 4 RFs: *N* = 36 (24.3%).

In Table 3 and Figure 3, for the CAD patients, there is significant difference among frequencies of HRCs with regard to the presence and number of risk factors (*p* = 0.040). Variability trends in the group of CAD patients regarding the number of risk factors show different pattern (V_1 RF_ = 45.89%, V_2 RF_ = 38.66%, V_3 RF_ = 33.55%, V_4 RF_ = 35.26%) versus variability in control sample (Table 3 and Figure 3).

Mean values of HRCs were significantly higher in CAD patients compared to control sample with 3 RFs (*p* < 0.001), while for the others, there were no significant differences (for 1 RF; 2 RFs; and 4 RFs, *p* > 0.05) (Table 3).

The presence of CAD in human individuals has a significant effect on the frequency distribution of HRCs (*p* = 0.004), as well as the number of risk factors (*p* = 0.009), while the presence of CAD and the number of RF interactions have no significant effect on the frequency distribution of HRCs (*p* = 0.057) (Table 3).

In Table 4, we presented an association between the frequencies of HRCs regarding the presence of CAD and number of risk factors versus controls. For the group with 2 RF, the presence of four HRCs (OR = 2.61) was significant predictor for the development of CAD, and for the group with 3 RF, the presence of five HRCs (OR = 5.33) was significant predictor for development of CAD as well (Table 4).

The results of ABO blood type frequencies (Table 5) demonstrated that A blood type (OR = 1.75) is significant predictor for CAD, while O blood type (OR = 0.43) was significantly associated with Controls.

## 4. Discussion

In our study, we have demonstrated that individuals with CAD have significantly higher proportion of present HTN and DM. Hypertension is shown by several studies as the most important risk factor for premature cardiovascular disease. It is more common among people than other major risk factors such as smoking, diabetes, and hyperlipidemia. These different cardiovascular risk factors have an additive effect on the likelihood of developing a coronary artery disease [28,29]. According to the meta-analysis of 102 prospective studies, DM is associated with a twice increased risk of CAD and mortality from the same [30]. Even though DM is usually present together with other risk factor for CAD such as HTN, hyperlipidemia comprising a known metabolic syndrome, DM itself brings an increased risk for CAD independently of the rest of risk factors [31].

Hyperlipidemia, as well smoking, showed no difference between CAD patients and the control sample. Since Serbia belongs to the group of countries which population has a very high frequency of smokers (41.4% of males and 37.6% females, according to the World Health Organization) [31]. This could be the reason for the absence of difference in smokers between the groups. The different impact of elevated lipid plasma levels on individuals with different CAD risk together with the need of management of lipid status dependable on the presence of other major risk factors, has led to the issue of rigorous and meticulous guidelines for lipid control and management. The lower acceptable value of total cholesterol and LDL-C might be the reason of no difference between the groups tested in our study, or the reason might lie in an earlier start of statin therapy, which has proven to lower coronary events and mortality when used for primary and secondary prevention [32,33,34].

Despite the fact that the modern medicine aims at finding a single gene responsible for CAD, it is becoming more obvious that many genes with various degree of influence participate in processes leading to development of CAD [27,35].

So far, for relatively a few morphophysiological processes gene locations are known. Thus, estimation of genetic homozygosity is a delicate and challenging task [8]. Previous studies have demonstrated that genetically-controlled biochemical processes expressing morphophysiological characteristics might be used to reveal the intrinsic changes in different groups of individuals with various conditions, implying a difference in their preferences or capabilities, as well as in their physiological and health capacities [9,10,11,12,13,14].

In our study, we have demonstrated existence of differences in the certain phenotype traits distribution between CAD patients and individuals from the control sample, assuming the possibility of changed predisposition existence for CAD patients. Such results imply to the correlation with different combinations of polygenes in these individuals. Further, it could be pointed out to the fact that there is the possibility of different, more specific phenotype existence for CAD patients versus individuals from the control sample. Therefore, our findings might argue that certain genes determining evaluated phenotypes that differ significantly between CAD patients and individuals from control sample could have, to the certain degree, the influence on the individual adaptability potential with the present CAD, to the specific environmental factors that could interfere during embryogenesis [36].

The degree of homozygosity markers evaluated in this study is shown to be significantly higher in the group of CAD patients versus individuals from the control sample. Along with decreased variability for tested genes in CAD patients group, it is obvious that population genetic difference exists between tested groups of individuals.

These results might be explained with the fact that higher degree of genetic homozygosity in CAD patients can lead to changes in genetic-hysiological homeostasis in such way that enables easier expression of CAD. Further, increased homozygosity degree might enable pleitropic effects of specific genes responsible for predisposition to CAD. Additionally, the state of increased genetic homozygosity could alter genetic load leading to the decrease in body immunity, enabling easier expression of CAD.

Considering the number of present RF along with the present CAD in studied individuals, we have demonstrated that their interaction does not have an influence on frequencies distribution of HRCs, while separately CAD and RF had significant effect on frequencies distribution of HRCs.

Additionally, our findings showed that as the number of RFs increased (1–4), the degree of genetic homozygosity varied, stressing out the changes in the genetic predispositions of tested groups of CAD patients. Such findings point out to the presence of the changes in genetic loads in CAD patients. This might suggest a presence of a complex polygenic differences among tested groups with different number of risk factors in CAD patients, enabling easier expression of CAD.

In the group of individuals with CAD, we noticed that A blood type frequency was significantly higher, while the frequency of O blood type was significantly lower compared to the controls. This is consistent with the literature which shows that non-O blood groups are associated with an increased risk of CAD [37]. In the systematic review and meta-analysis of Chen et al. [37], it was stated that the possible mechanism of the relationship between O blood type and CAD refers to the fact that individuals with non-O blood types have relatively higher levels of von Willebrand factor (vWF) and factor VIII. Aside from this, it was noticed as well that individuals with A blood type have higher levels of serum total cholesterol [37].

There are several limitations for this study. It is conducted on individuals only from the Serbian population. Due to the fact that specific socio-economic and genetic variations may exist among different populations, further studies conducted on different populations are needed. Another limiting factor includes the number of patients, thus, further studies on larger sample are advised.

In conclusion, given all stated above, we have demonstrated higher degree of recessive homozygosity in CAD patients versus individuals in control sample, and changed variability in the degree of recessive homozygosity as the number of tested RFs increases. Such observation shows that, to the certain degree, different genes in different proportion could be manifested and responsible in the processes governing the development and the degree of expression of CAD. Additionally, we have shown that A blood type might be considered as a potential predictor of CAD.

Despite the numerous population genetic studies previously conducted [8,9,10,11,12,13,14,15,16,20,21,22], establishing expected phenotype variations in humans is still a challenging task. Our findings are in line with such statement, since we have demonstrated the presence of differences in such variations between evaluated groups. These findings might have potential in broader understanding of multifactorial etiology of CAD, and conducted methodology of morphophysiological traits testing (HRC test) could be proposed as an additional screening method for CAD in humans.

It should be worth mentioning, as well, that the genome wide association study (GWAS) and next generation sequencing in patients with CAD could identify regions with loss of heterozygosity and/or copy number variation adding an additional information on CAD inheritance along with the evaluation of the degree of genetic homozygosity.

## Figures and Tables

**Table 1:** Demographic and risk factor frequencies.

Parameters	Control Sample *N* = 156	CAD Patients *N* = 148	*p*
Male *n* (%)	80 (51.3)	96 (64.9)	>0.05 *
Female *n* (%)	76 (48.7)	52 (35.1)	
HTN *n* (%)	88 (56.4)	142 (95.9)	<0.01 *
DM *n* (%)	32 (20.5)	82 (55.4)	<0.01 *
HLP *n* (%)	100 (64.1)	106 (71.6)	>0.05 *
Smoking *n* (%)	68 (43.6)	78 (52.7)	>0.05 *

CAD: Coronary artery disease; HTN: Hypertension; DM: Diabetes mellitus; HLP: Hyperlipidemia; * *χ*^2^ test.

**Table 2:** Frequencies of homozygously-recessive characteristics among patients with CAD and individuals of the control sample.

Homozygously Recessive Characteristics	Control Sample *N* = 156, *n* (%)	CAD Patients *N* = 148, *n* (%)	*χ* ^2^	OR (95% CI)
Blond Hair	45 (28.8)	36 (24.3)	0.703	0.79 (0.48–1.32)
Straight Hair	92 (59.0)	74 (50.0)	1.373	0.70 (0.44–1.09)
Double Hair Whorl	15 (9.6)	5 (3.4)	4.004 *	0.33 * (0.12–0.93)
Opposite Hair Whorl Orientation	21 (13.5)	29 (19.6)	2.756	1.57 (0.85–2.89)
Soft Hair	57 (36.5)	43 (29.1)	1.500	0.71 (0.44–1.15)
Attached Ear Lobe	18 (11.5)	12 (8.1)	1.005	0.68 (0.31–1.46)
Ear Without Darwinian notch	5 (3.2)	19 (12.8)	28.800 **	4.45 ** (1.62–12.25)
Blue Eyes	32 (20.5)	67 (45.3)	30.002 **	3.21 ** (1.93–5.32)
Speaking deficiency-gutorral “r”	3 (1.9)	9 (6.1)	9.284 **	3.30 (0.88–12.44)
Inability to Transversally Tongue Roll	40 (25.6)	76 (51.4)	26.002 **	3.06 ** (1.89–4.96)
Inability to Longitudinally Tongue Roll	20 (12.8)	48 (32.4)	30.013 **	3.26 ** (1.82–5.84)
Right Thumb over Left Thumb	84 (53.8)	82 (55.4)	0.048	1.06 (0.68–1.67)
Top Joint of the Thumb >45°	35 (22.4)	14 (9.5)	7.429 **	0.36 ** (0.19–0.70)
Hypermobility of proximal thumb joint	9 (5.8)	12 (8.1)	0.912	1.44 (0.59–3.53)
Proximal thumb extensibility	28 (17.9)	54 (36.5)	19.327 **	2.63 ** (1.55–4.45)
Left-handedness	17 (10.9)	9 (6.1)	2.114	0.53 (0.23–1.23)
Index finger longer than the ring finger	64 (41.0)	42 (28.4)	3.872 *	0.57 * (0.35–0.92)
∑*χ*^2^ = 169.144 **				

CAD: Coronary artery disease; * *p* < 0.05; ** *p* < 0.01.

**Table 3:** Distribution of mean HRC values based on the study of 17 qualitative morpho-physiological traits in CAD patients and control sample regarding presence and number of risk factors.

RF	HRC (MV ± SD)		(*p*) **	HRC, V (%)	
	Control Sample	CAD Patients		Control Sample	CAD Patients
0	4.63 ± 1.38	0	-	29.81	0
1	4.13 ± 1.68	4.75 ± 2.18	0.569	39.47	45.89
2	3.48 ± 1.82	3.88 ± 1.50	0.211	52.30	38.66
3	3.15 ± 1.54	4.65 ± 1.56	<0.001	48.89	33.55
4	3.92 ± 1.31	3.97 ± 1.40	0.920	33.42	35.26
(*p*) *	(0.007)	(0.040)	-	-	-
A ***	0.004				
B ***	0.009				
C ***	0.057				

RF: Risk factors; HRC: Homozygously-recessive characteristics; CAD: Coronary artery disease; MV: Mean value; SD: Standard deviation; V: Variability; * Unifactorial ANOVA; ** Mann-Whitney U test; *** Two-way ANOVA; A: Presence of CAD and frequencies distribution of HRCs; B: number of RF and frequencies distribution of HRCs; C: presence of CAD and number of RFs interaction, and frequencies distribution of HRCs.

**Table 4:** Association between frequencies of homozygous recessive characteristics regarding the presence of coronary artery disease and number of risk factors versus controls.

Number of HRC	Controls/CAD, OR (95% CI)			
	1 RF	2 RF	3 RF	4 RF
0	-	-	-	-
1	1.36 (0.11–16.58)	0.35 (0.07–1.81)	0.20 (0.02–2.03)	-
2	3.22 (0.55–18.85)	0.70 (0.23–1.89)	0.48 (0.12–1.95)	0.45 (0.07–3.11)
3	0.60 (0.11–3.38)	0.88 (0.32–2.45)	0.44 (0.15–1.25)	1.92 (0.36–10.36)
4	1.40 (0.22–8.86)	2.61 * (0.99–6.88)	0.58 (0.20–1.66)	0.62 (0.16–2.37)
5	0.23 (0.03–2.07)	0.90 (0.34–2.41)	5.33 * (1.12–25.39)	2.20 (0.24–20.39)
6	0.64 (0.06–6.35)	1.73 (0.28–10.84)	3.10 (0.80–11.95)	0.63 (0.10–3.94)
7	6.20 (0.51–75.84)	1.13 (0.22–5.90)	-	-
8	-	-	-	-

Note: * *p* < 0.05.

**Table 5:** Frequencies of ABO blood types among CAD patients and controls.

Blood Type	Controls *N* = 156, *n* (%)	CAD patients *N* = 148, *n* (%)	OR (95% CI)
A	51 (32.7)	68 (45.9)	1.75 * (1.10–2.77)
B	31 (19.9)	44 (29.7)	1.70 (1.01–2.89)
O	58 (37.1)	30 (20.3)	0.43 ** (0.26–0.72)
AB	16 (10.3)	6 (4.1)	0.37 (0.14–0.97)

Note: * *p* < 0.05; ** *p* < 0.01.
